# Supplementary material for: Molecular and electronic structure of terminal and alkali metal-capped uranium(V) nitride complexes
Source: Nat Commun. 2016 Dec 20;7:13773. doi: 10.1038/ncomms13773 (PMC5187438; doi:10.1038/ncomms13773)
Supplement: Supplementary Information — Supplementary Figures, Supplementary Tables, Supplementary Notes, Supplementary Methods and Supplementary References. [file ncomms13773-s1.pdf]

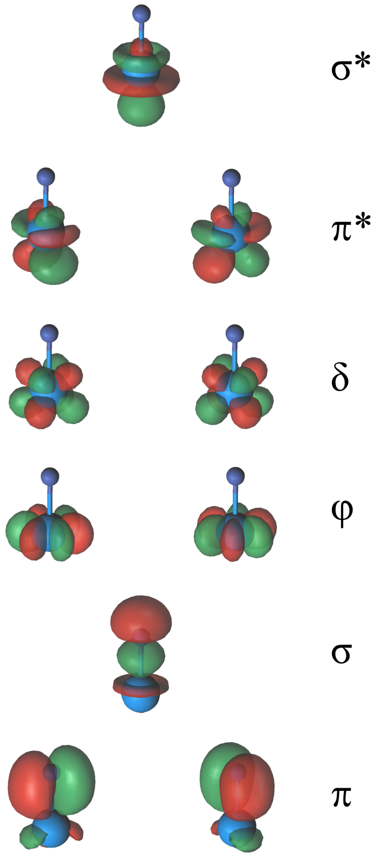

Supplementary Figure 1. MOs for the $[UN]^{2+}$ species from *ab initio* calculations. U = light blue (lower), N = dark blue (upper).

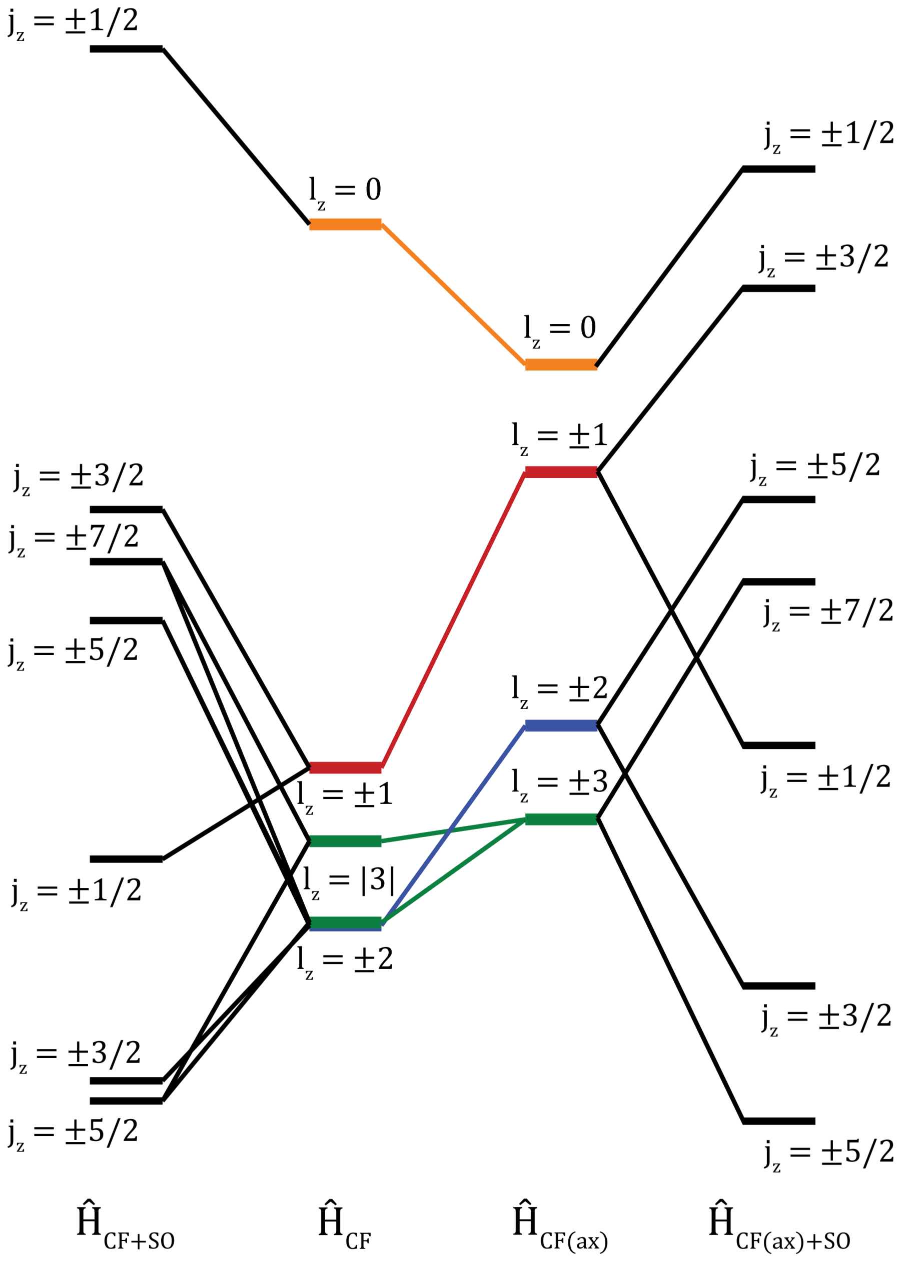

Supplementary Figure 2. Simplified diagram of states for $[U(NH_3)(NH_2)_3(N)]^-$ and $[UN]^{2+}$ model complexes, illustrating how different ground states can arise from the interplay of CF and SOC. States are those arising from the D_3 symmetric $\hat{H}_{CF}$, appropriate for symmetrised $[U(NH_3)(NH_2)_3(N)]^-$ (centre left), and the $C_{\infty v}$ symmetric $\hat{H}_{CF(ax)}$, appropriate for $[UN]^{2+}$ (centre right), and the effect of $\hat{H}_{SO}$ on both (extreme left and right, respectively). Energies are taken from *ab initio* calculations for $[U(NH_3)(NH_2)_3(N)]^-$ and $[UN]^{2+}$, respectively.

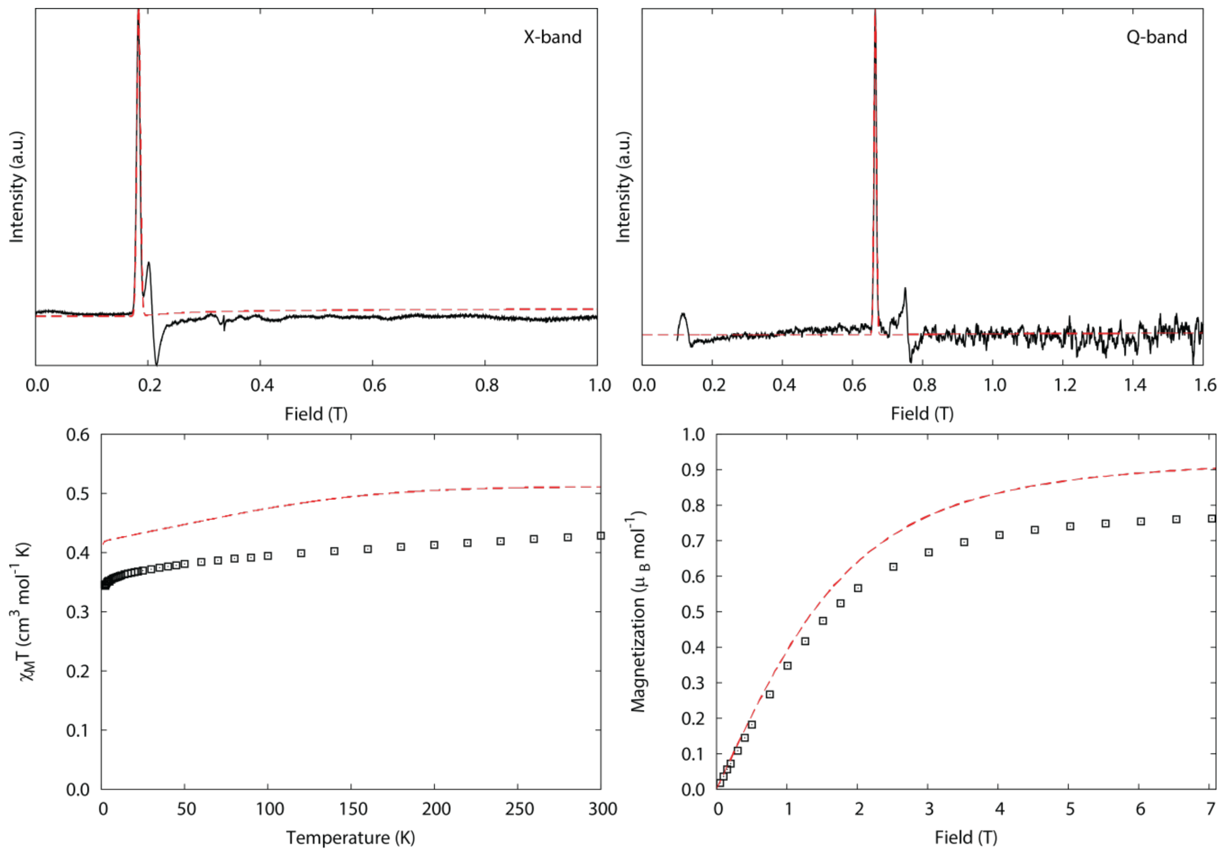

Supplementary Figure 3. Experimental (black curves and points) and fitted (red dashed curves) EPR spectra, $\chi_M T$ and magnetisation for **3K**. EPR spectra recorded at 5 K with frequencies of 9.393423 and 34.0321 GHz, respectively. The secondary feature at *ca.* 0.2 T at X-band, and *ca.* 0.75 T at Q-band, are due to polycrystalline effects. $\chi_M T$ and magnetisation recorded at 0.1 T and 1.8 K, respectively. Magnetic data shown per uranium.

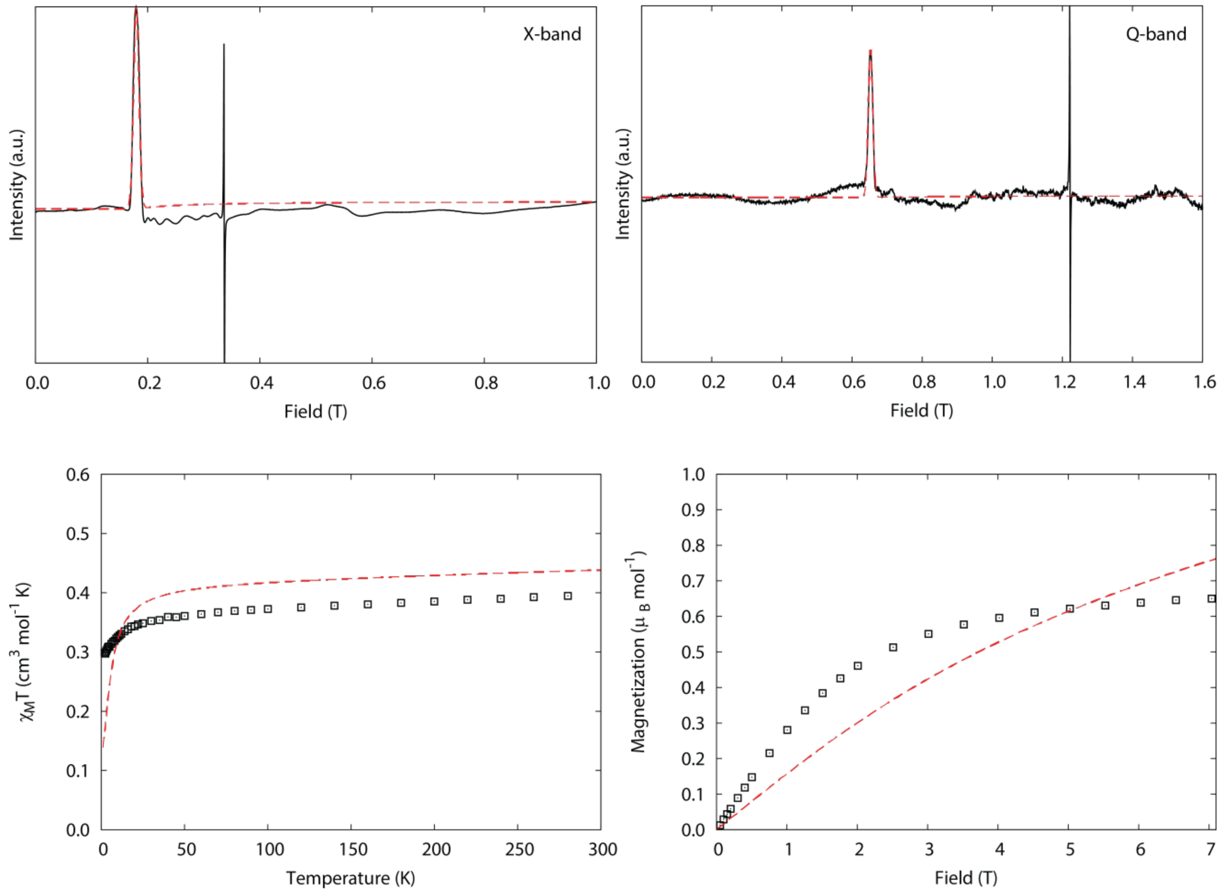

Supplementary Figure 4. Experimental (black curves and points) and fitted (red dashed curves) EPR spectra, $\chi_M T$ and magnetisation for **3Rb**. EPR spectra recorded at 5 and 6 K with frequencies of 9.3943 and 34.1114 GHz, respectively. $\chi_M T$ and magnetisation recorded at 0.1 T and 1.8 K, respectively. Magnetic data shown per uranium.

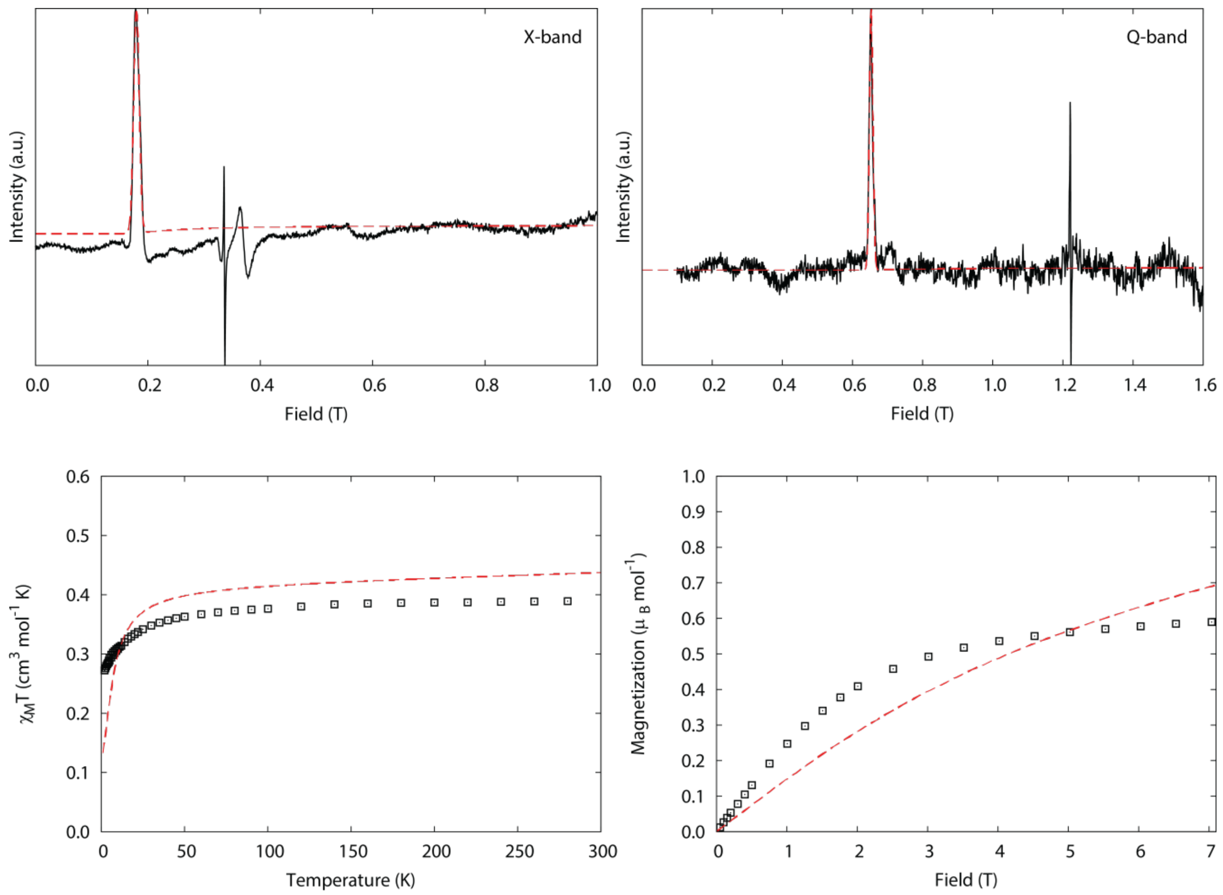

Supplementary Figure 5. Experimental (black curves and points) and fitted (red dashed curves) EPR spectra, $\chi_M T$ and magnetisation for **3Cs**. EPR spectra recorded at 5 and 6 K with frequencies of 9.3852359 and 34.1274 GHz, respectively. $\chi_M T$ and magnetisation recorded at 0.1 T and 1.8 K, respectively. Magnetic data shown per uranium.

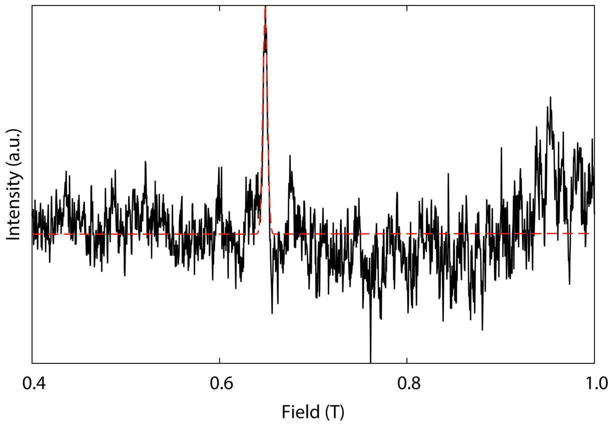

Supplementary Figure 6. Experimental (black curve) and fitted (red dashed curve) Q-band EPR spectrum for **4Na**, recorded at 5 K and 34.277 GHz.

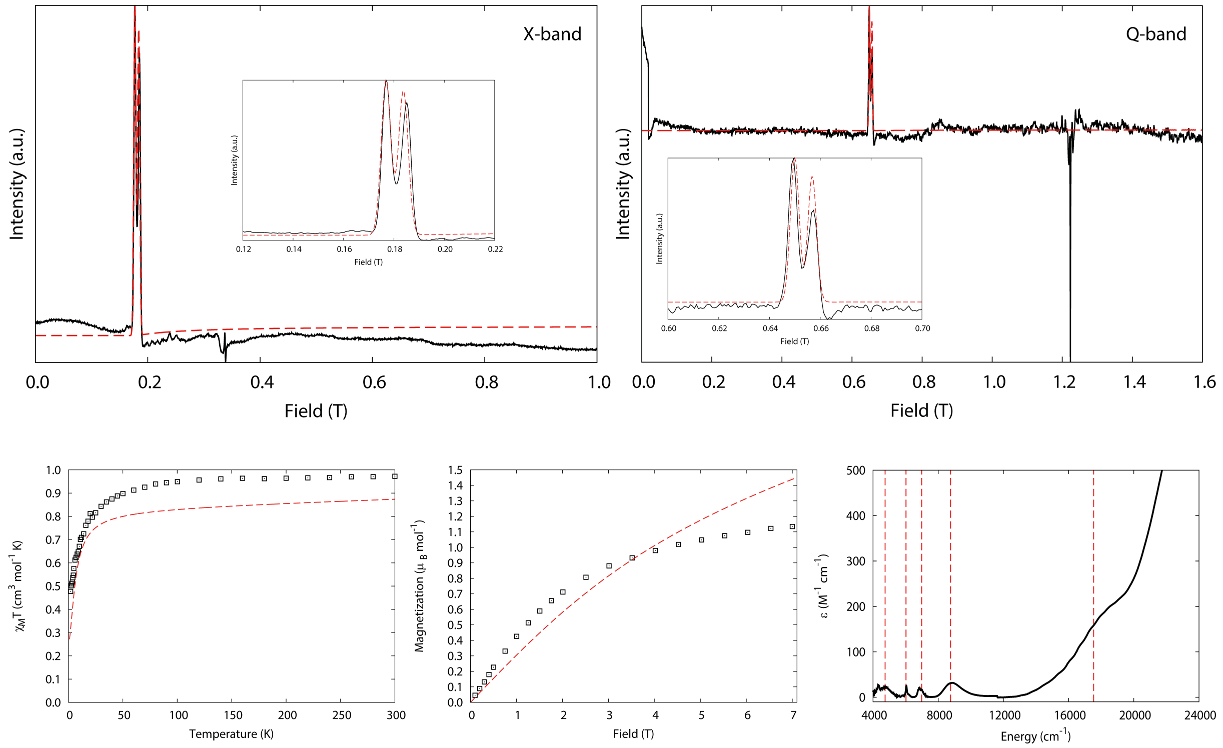

Supplementary Figure 7. Experimental (black curves and points) and fitted (red dashed curves) EPR spectra, $\chi_M T$, magnetisation and UV/Vis/NIR data for **4K**. EPR spectra recorded at 10 and 5 K with frequencies of 9.408141 and 33.0863 GHz, respectively. $\chi_M T$, magnetisation and UV/Vis/NIR data recorded at 0.1 T, 1.8 K and room temperature, respectively. Magnetic data shown for a uranium dimer. Dipolar interactions are also responsible for the additional spectral splitting of the $g_z \approx 3.7$ peak. **4K** is the only monometallic complex that crystallises in space group P−1, making all molecules collinear in the solid state. The nearest U···U distances are 9.56 Å, with an angle of 33.3° between U≡N and U···U vectors, giving a point-dipole interaction of j_{zz} ca. +0.004 cm^{-1} which compares well with the experimental j_{zz} = +0.0066 cm^{-1}.

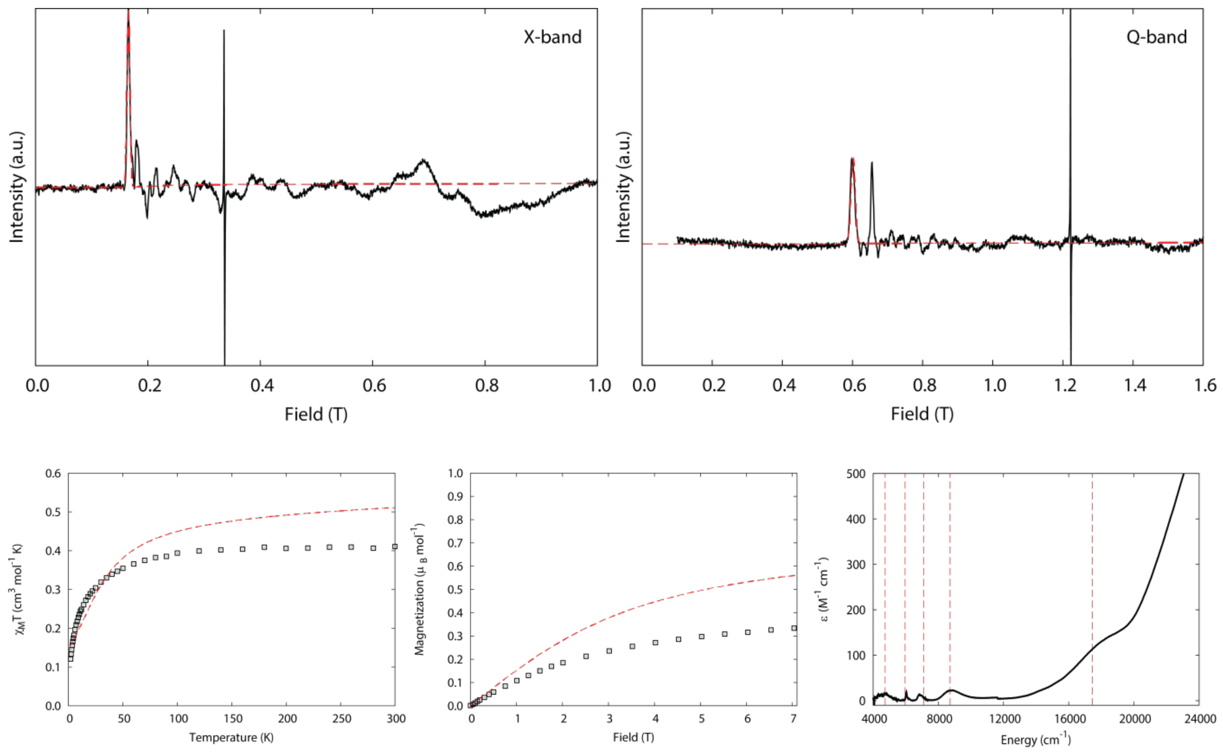

Supplementary Figure 8. Experimental (black curves and points) and fitted (red dashed curves) EPR spectra, $\chi_M T$, magnetisation and UV/Vis/NIR data for **4Rb**. EPR spectra recorded at 8 and 6 K with frequencies of 9.37517 and 34.118 GHz, respectively. The features above *ca.* 0.2 T at X-band, and *ca.* 0.7 T at Q-band, are due to polycrystalline effects and not exchange coupling – the lowest field feature represents the true g_z value and thus this feature has been fitted. $\chi_M T$, magnetisation and UV/Vis/NIR data recorded at 0.1 T, 1.8 K and room temperature, respectively.

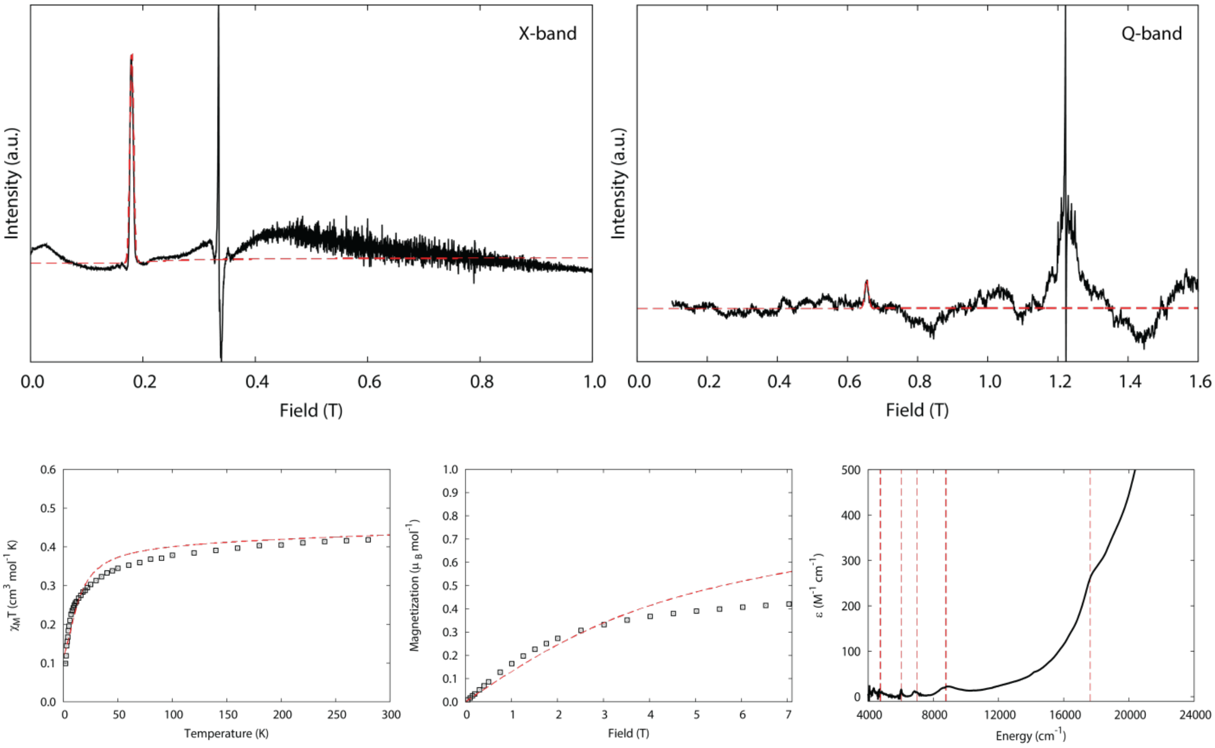

Supplementary Figure 9. Experimental (black curves and points) and fitted (red dashed curves) EPR spectra, $\chi_M T$, magnetisation and UV/Vis/NIR data for **4Cs**. EPR spectra recorded at 5 K with frequencies of 9.3873 and 34.1009 GHz, respectively. $\chi_M T$, magnetisation and UV/Vis/NIR data recorded at 0.1 T, 1.8 K and room temperature, respectively.

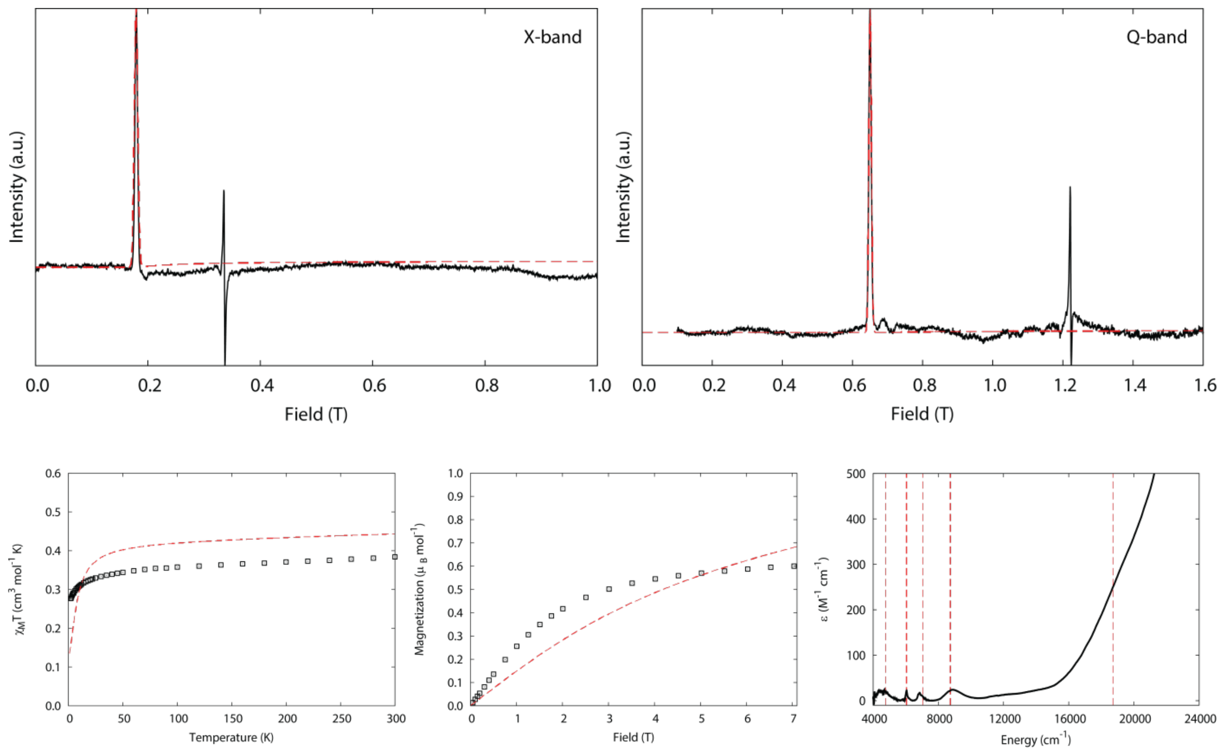

Supplementary Figure 10. Experimental (black curves and points) and fitted (red dashed curves) EPR spectra, $\chi_M T$, magnetisation and UV/Vis/NIR data for **5Li**. EPR spectra recorded at 5 and 6 K with frequencies of 9.38207 and 34.1176 GHz, respectively. $\chi_M T$, magnetisation and UV/Vis/NIR data recorded at 0.1 T, 1.8 K and room temperature, respectively.

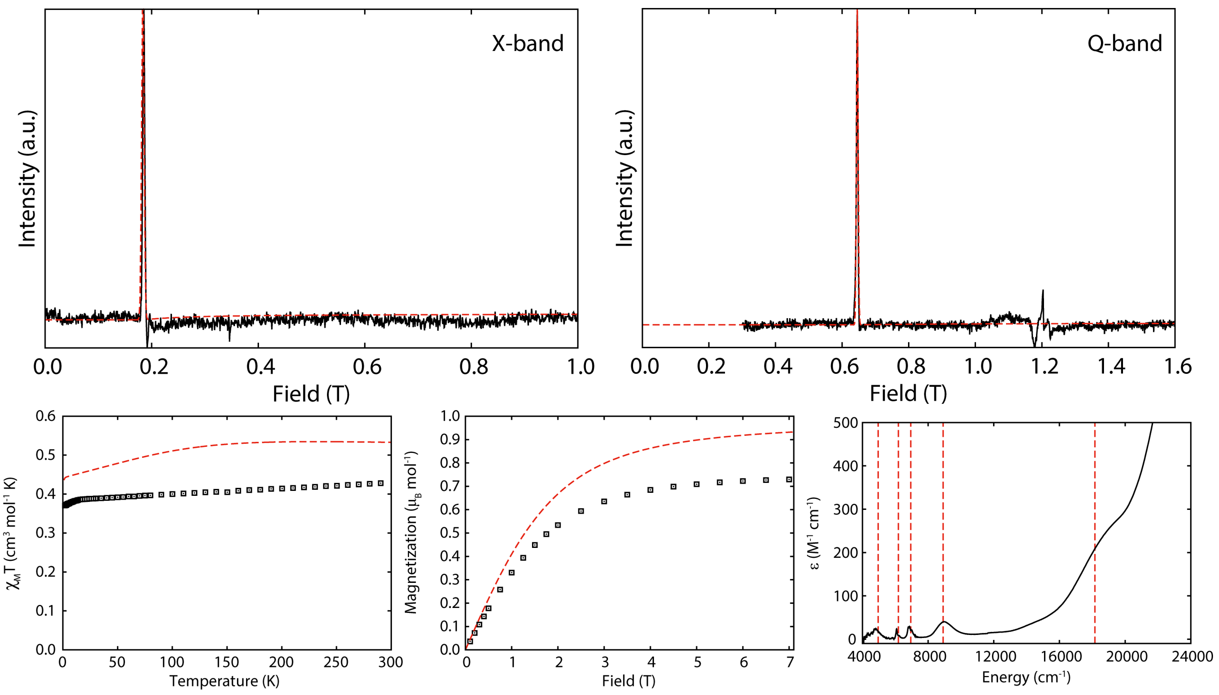

Supplementary Figure 11. Experimental (black curves and points) and fitted (red dashed curves) EPR spectra, $\chi_M T$, magnetisation and UV/Vis/NIR data for **5Na**. EPR spectra recorded at 5 K with frequencies 9.63793 and 33.937 GHz, respectively. $\chi_M T$, magnetisation and UV/Vis/NIR data recorded at 0.1 T, 1.8 K and room temperature, respectively.

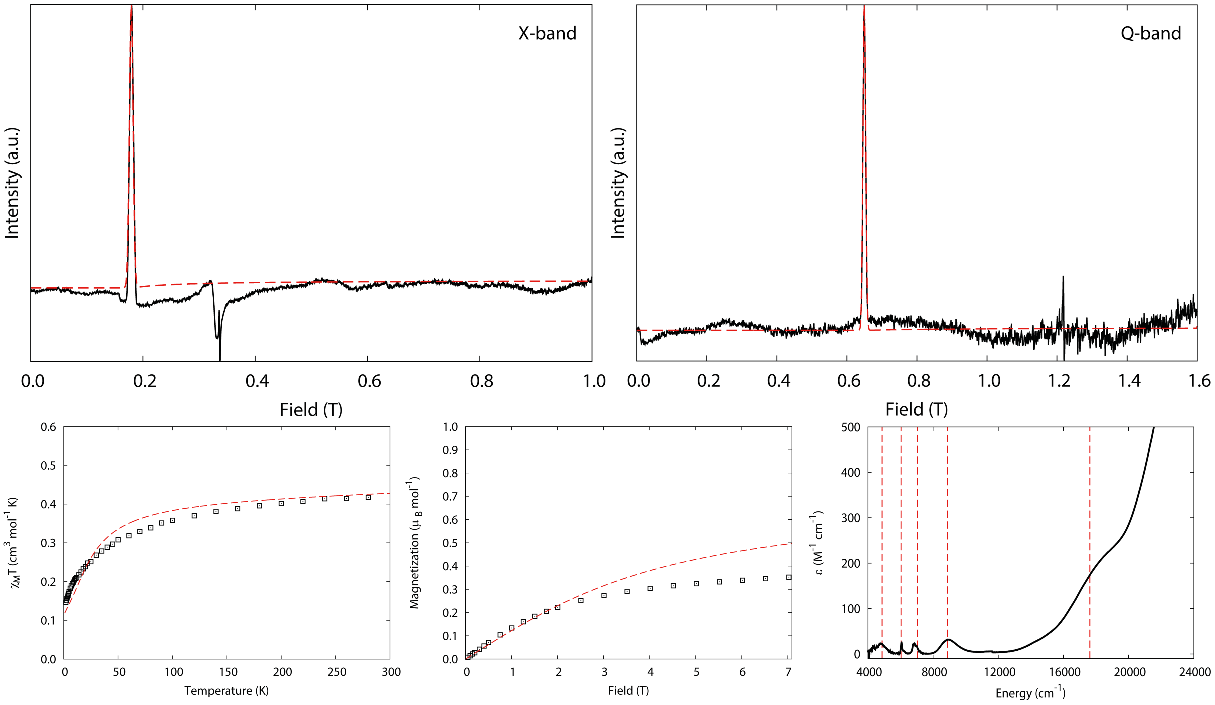

Supplementary Figure 12. Experimental (black curves and points) and fitted (red dashed curves) EPR spectra, $\chi_M T$, magnetisation and UV/Vis/NIR data for **5K**. EPR spectra recorded at 5 and 8 K with frequencies of 9.38237 and 33.043 GHz, respectively. $\chi_M T$, magnetisation and UV/Vis/NIR data recorded at 0.1 T, 1.8 K and room temperature, respectively.

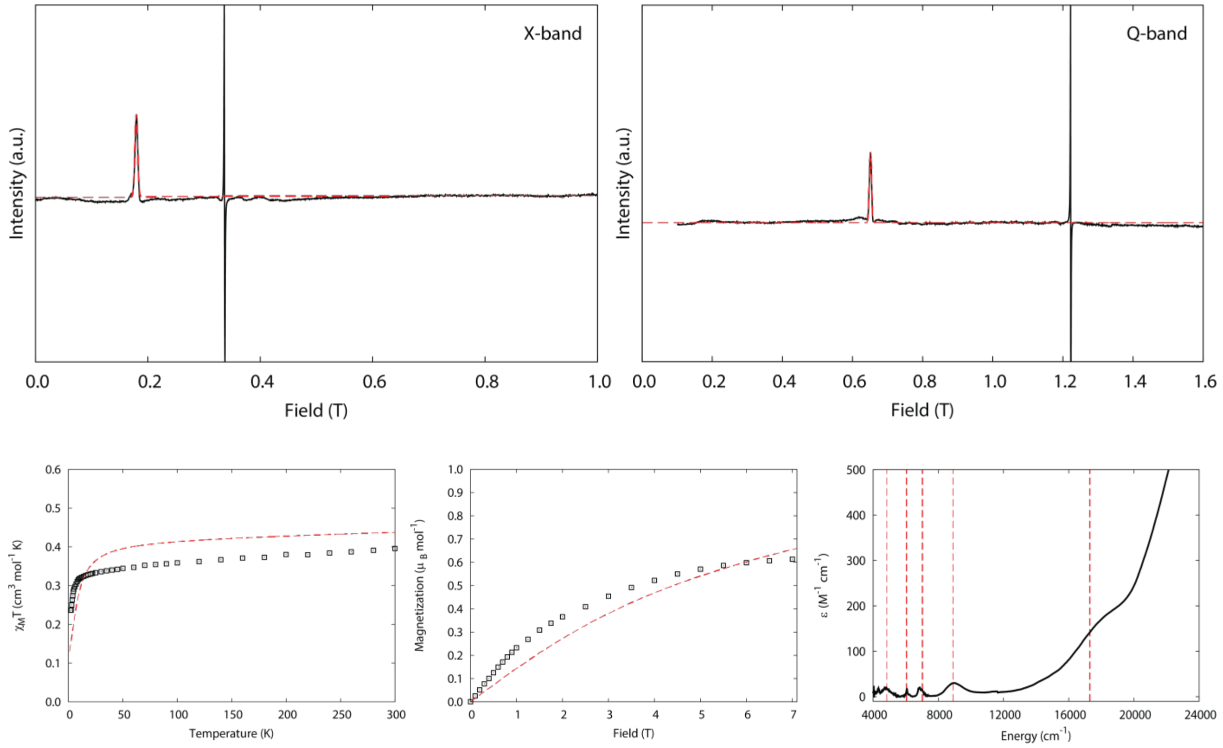

Supplementary Figure 13. Experimental (black curves and points) and fitted (red dashed curves) EPR spectra, $\chi_M T$, magnetisation and UV/Vis/NIR data for **5Rb**. EPR spectra recorded at 5 and 6 K with frequencies of 9.38474 and 34.1145 GHz, respectively. $\chi_M T$, magnetisation and UV/Vis/NIR data recorded at 0.1 T, 1.8 K and room temperature, respectively.

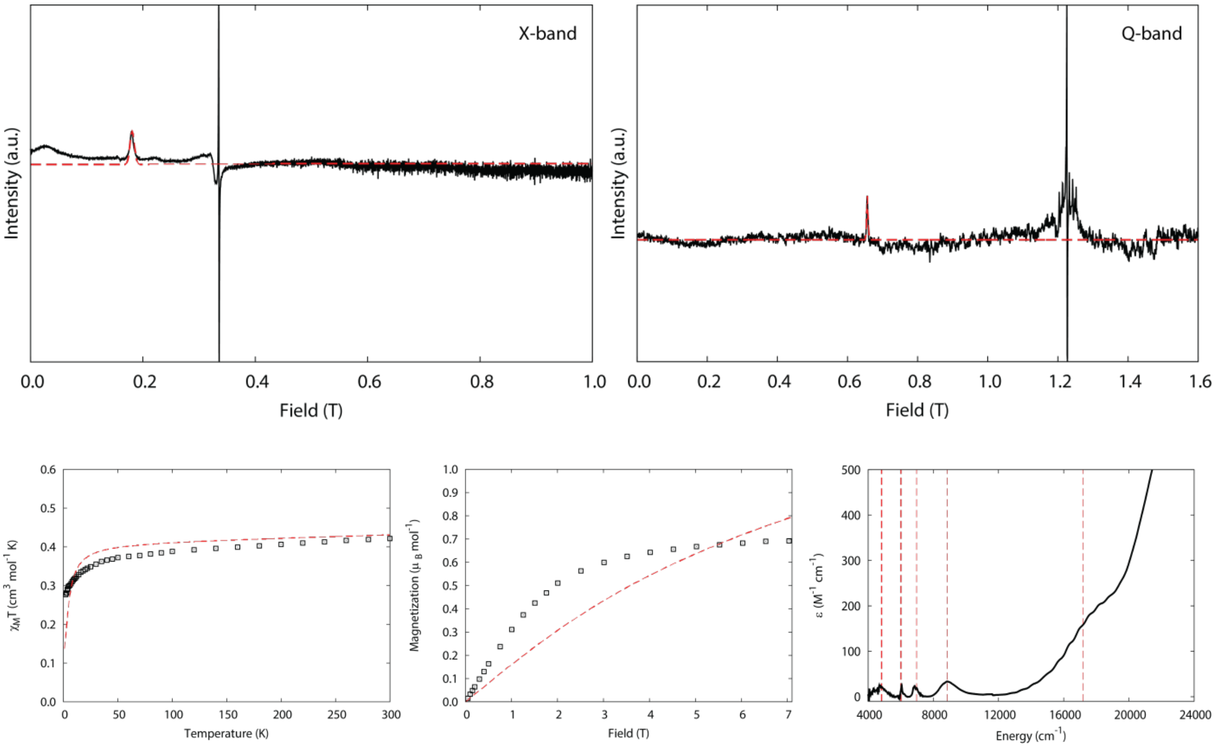

Supplementary Figure 14. Experimental (black curves and points) and fitted (red dashed curves) EPR spectra, $\chi_{\mathrm{M}}T$, magnetisation and UV/Vis/NIR data for **5Cs**. EPR spectra recorded at 5 K with frequencies of 9.3959 and 34.10096 GHz, respectively. $\chi_{\mathrm{M}}T$, magnetisation and UV/Vis/NIR data recorded at 0.1 T, 1.8 K and room temperature, respectively.

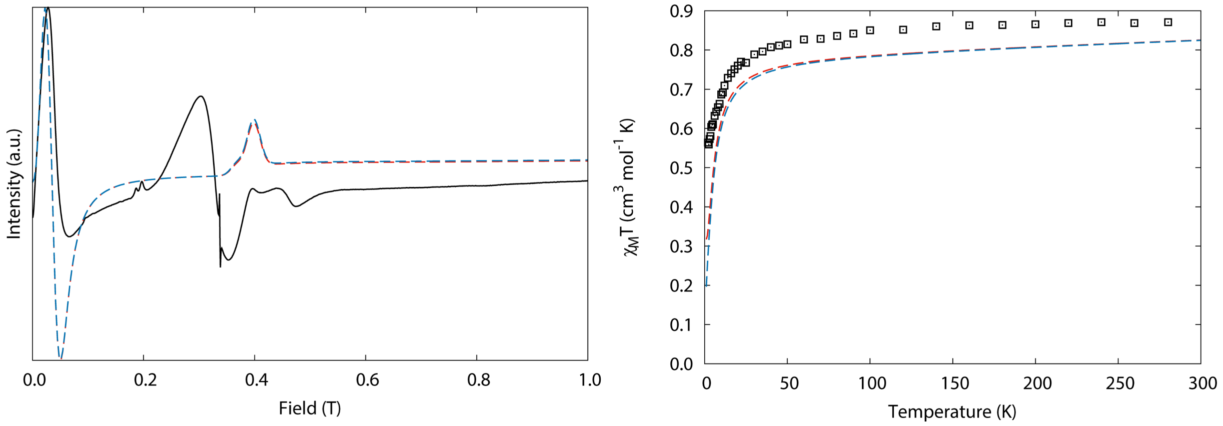

Supplementary Figure 15. Experimental (black curve and points) and fitted (using Hamiltonian (2) for **3Na**, red dashed curve j_{zz} = +0.7678 cm^{-1}, blue dashed curve j_{zz} = –0.7678 cm^{-1}) Q-band EPR spectrum and $\chi_M T$ for **3Na**. EPR spectra recorded at 7 K and 33.0787 GHz. $\chi_M T$ recorded under a 0.1 T. All data given for a uranium dimer.

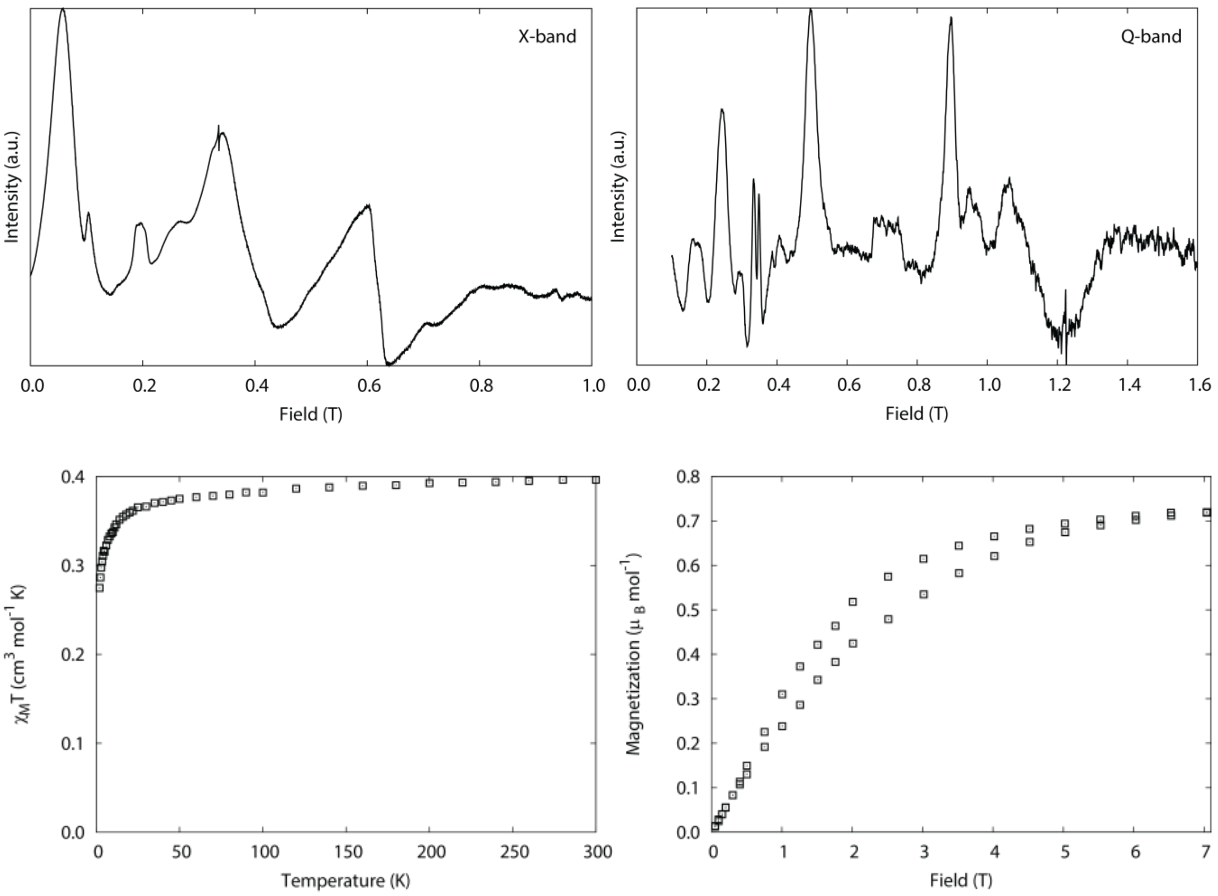

Supplementary Figure 16. Experimental EPR spectra, $\chi_M T$ and magnetisation for **3Li**. EPR spectra recorded at 5 K with frequencies of 9.3889 and 34.277 GHz, respectively. $\chi_M T$ and magnetisation recorded at 0.1 T and 1.8 K, respectively. Magnetic data given per uranium.

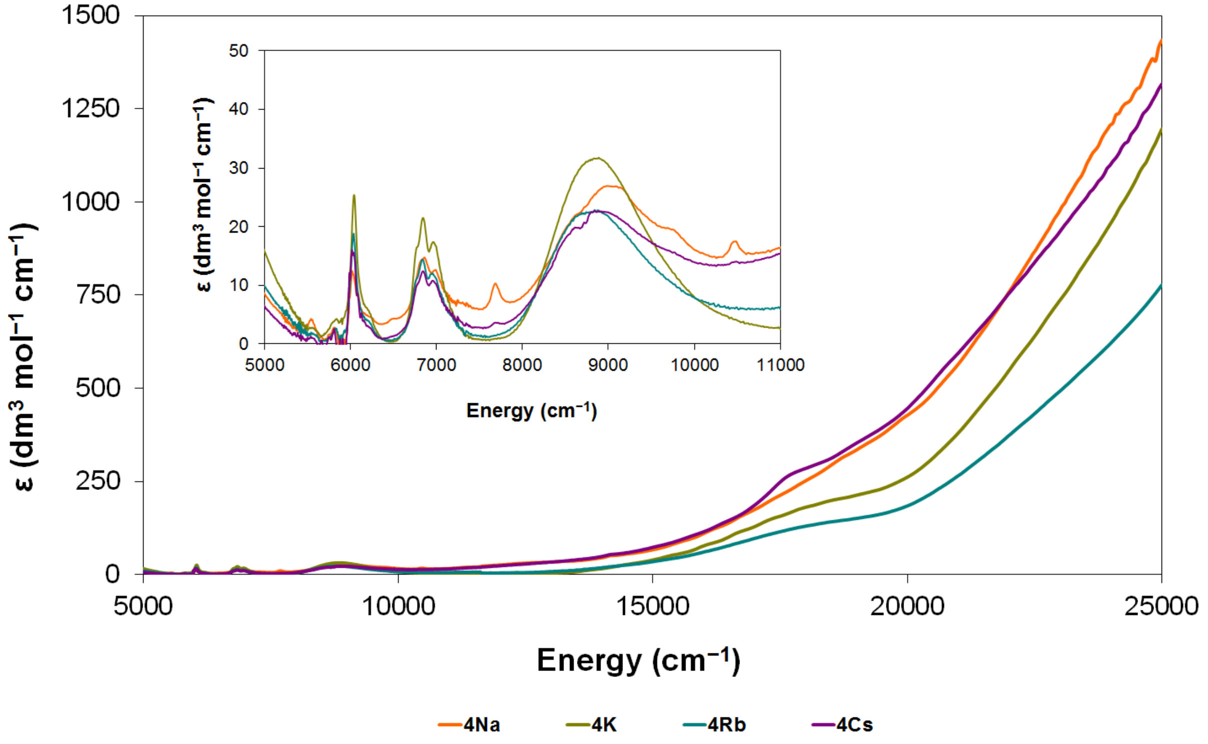

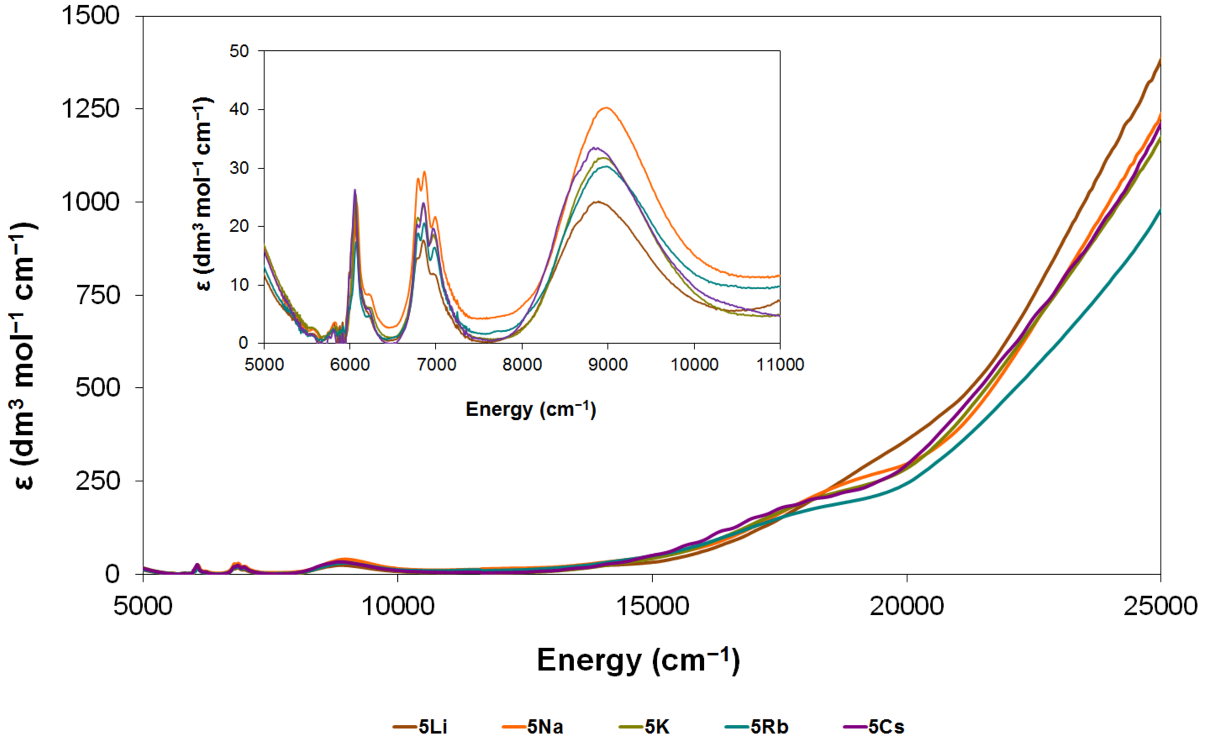

Supplementary Figure 17. Top: UV/Vis/NIR spectra of the SIP series **4M**. Bottom: UV/Vis/NIR spectra of the SIP series **5M**. All spectra were recorded in toluene in a 1 mm quartz cuvette at 298 K.

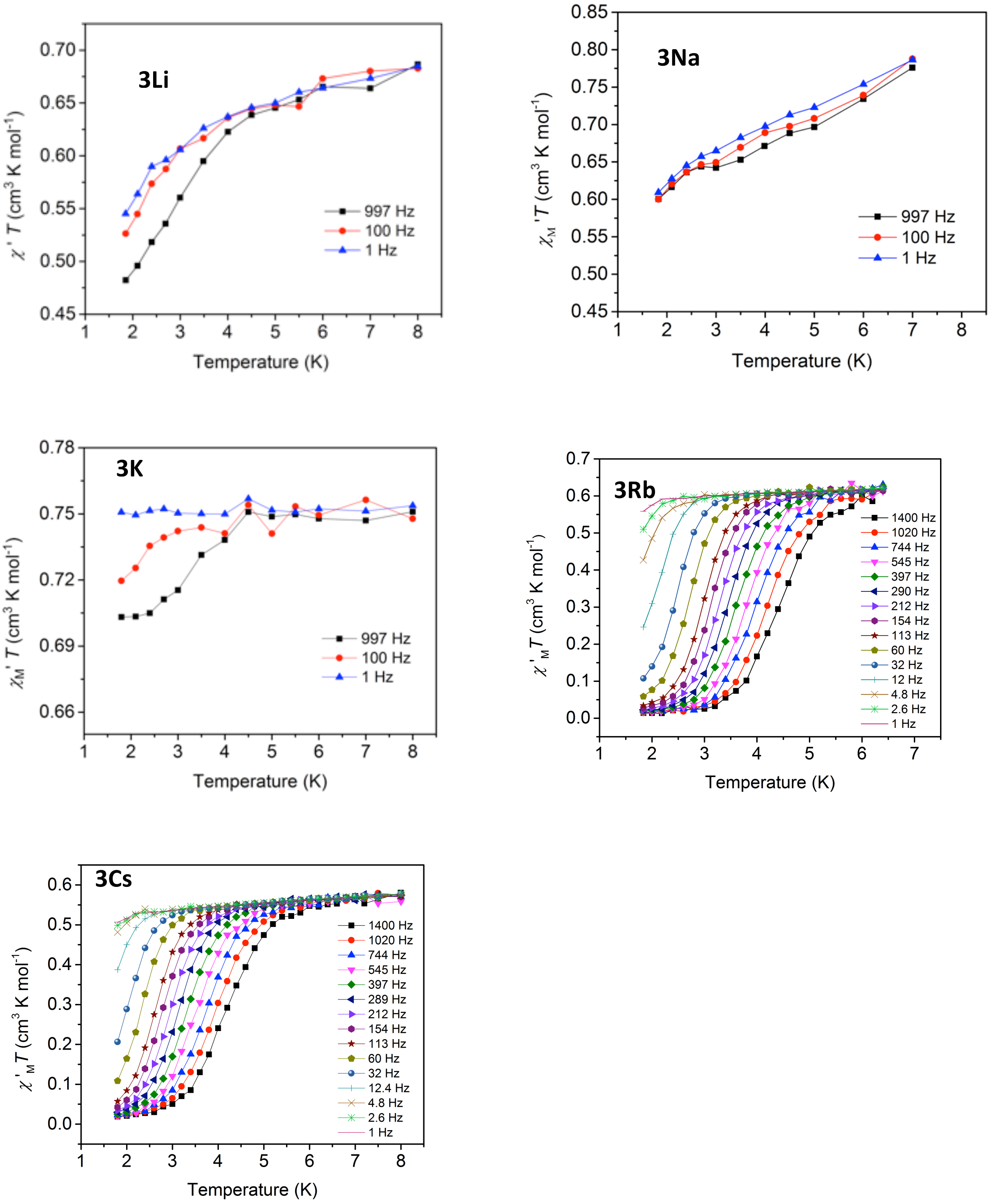

Supplementary Figure 18. Experimental $\chi'T(T)$ for **3Li** (0.1 T), **3Na** (0 T), **3K** (0 T), **3Rb** (0.1 T), **3Cs** (0.1 T applied dc field).

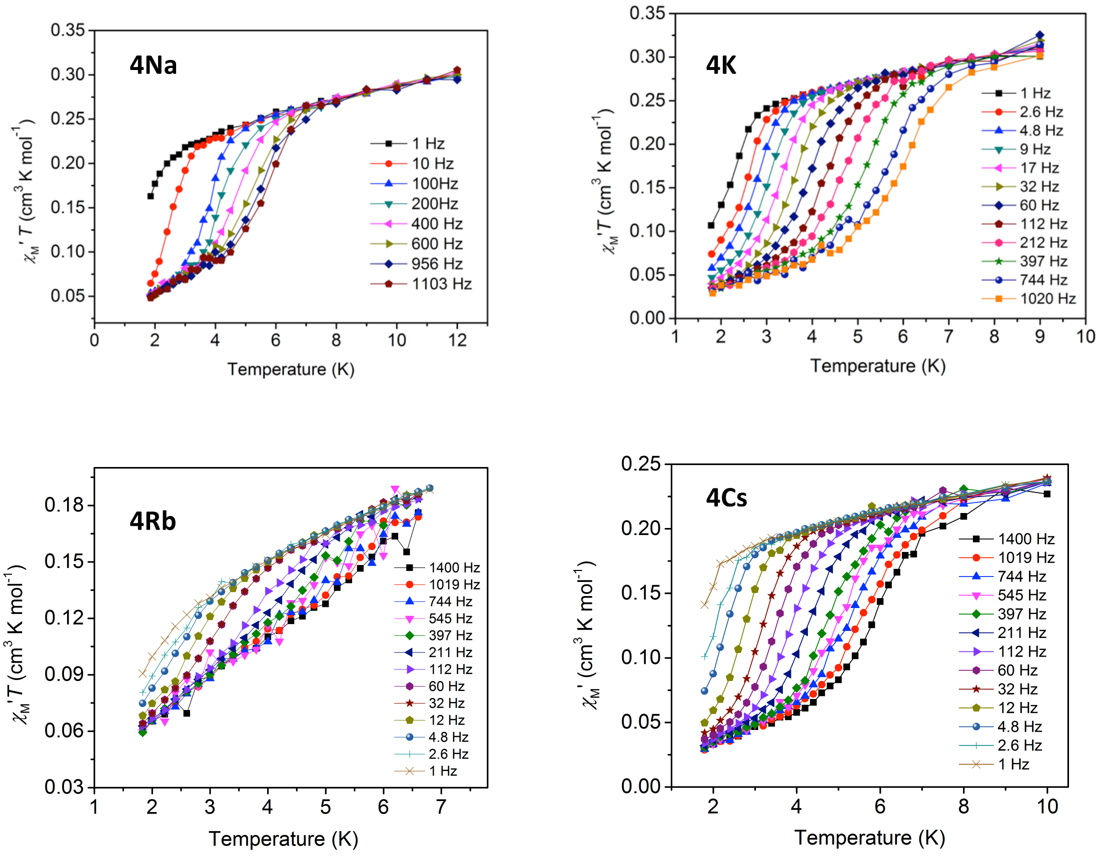

Supplementary Figure 19. Experimental $\chi'T(T)$ for **4Na** (0.1 T), **4K** (0.1 T), **4Rb** (0.1 T), **4Cs** (0.1 T applied dc field).

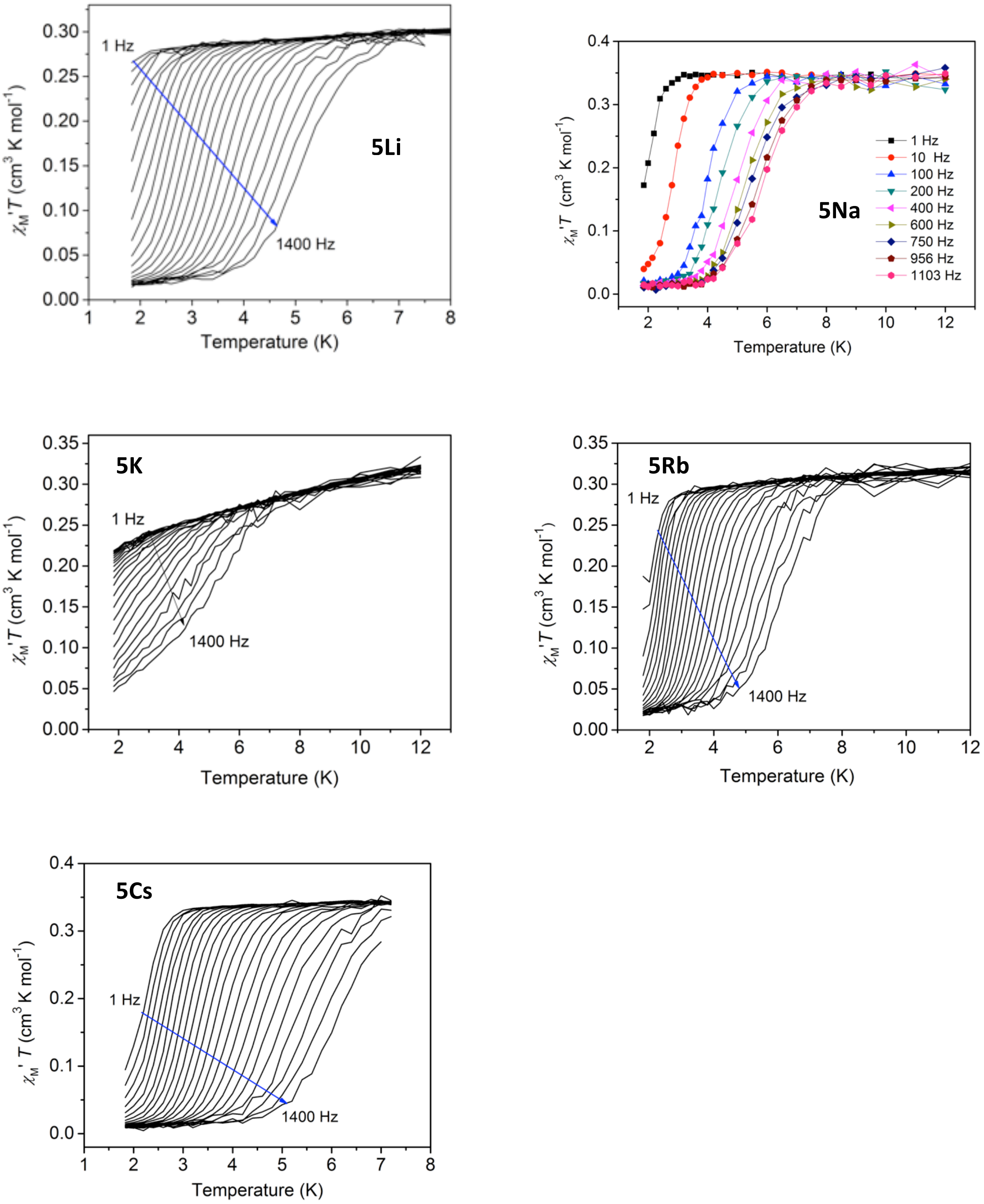

Supplementary Figure 20. Experimental $\chi'T(T)$ for **5Li** (0.1 T), **5Na** (0.1 T), **5K** (0.1 T), **5Rb** (0.1 T), **5Cs** (0.1 T applied dc field).

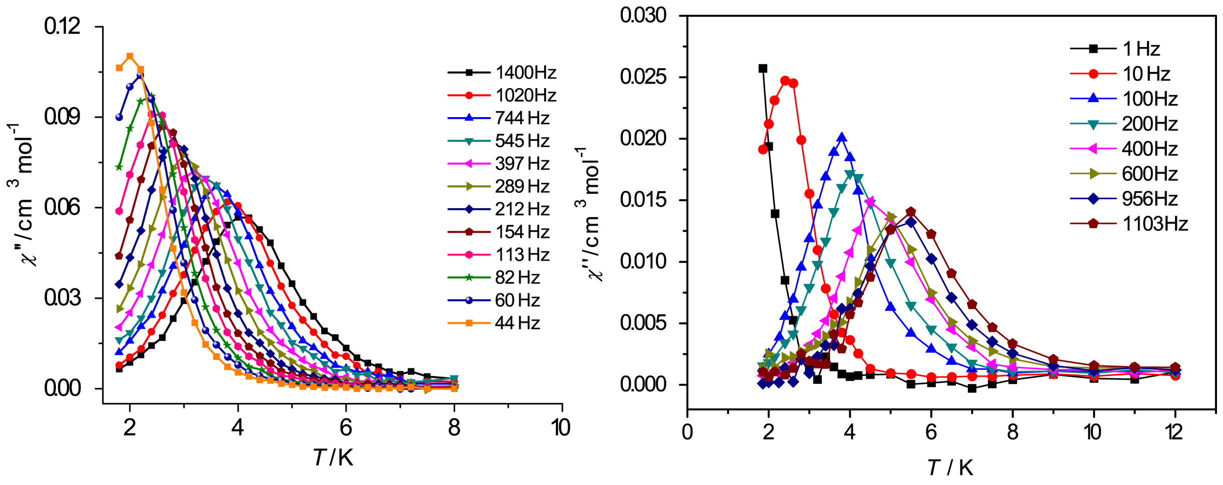

Supplementary Figure 21. Experimental $\chi''(T)$ for **3Cs** and **4Na** recorded under a 0.1 T field. Lines just join the points.

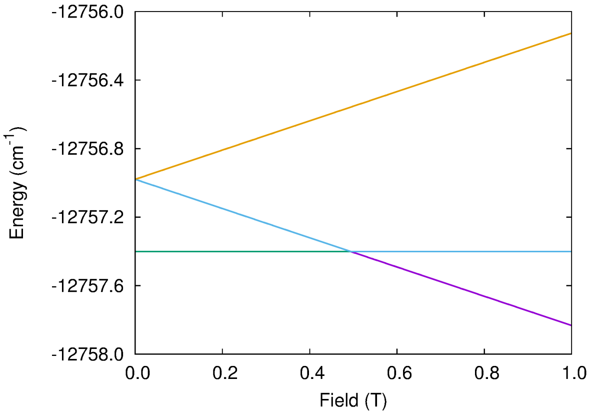

Supplementary Figure 22. Lowest lying exchange states of **3Na** as a function of magnetic field along the z-axis, calculated from the exchange coupled CF and SOC Hamiltonian, Equation 2 (main text), where $j_{zz} = -0.7678$ cm^{-1}.

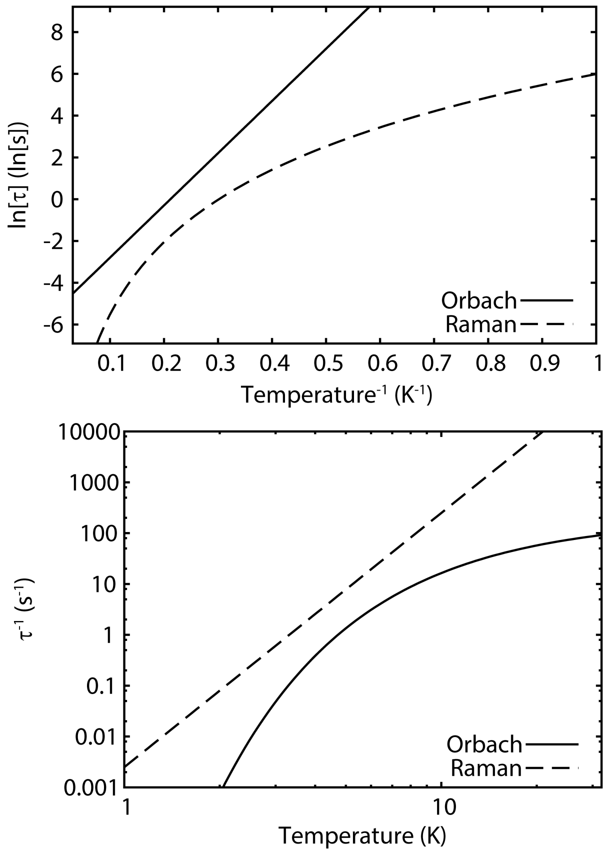

Supplementary Figure 23. Theoretical temperature dependence for the Orbach/TA-QTM and Raman relaxation mechanisms, using hypothetical parameters U_{eff} = 25 K, τ_0 = 0.005 s, C = 0.0025 K^{-5} s^{-1} and n = 5. Both plots cover the same range of τ and T.

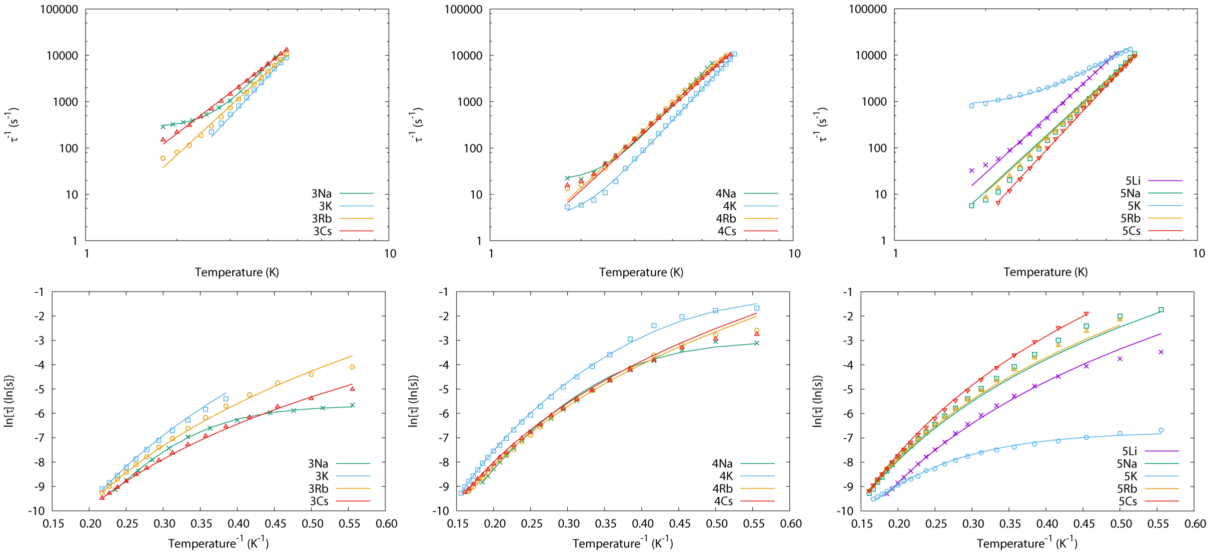

Supplementary Figure 24. Experimental (points) and fitted (curves) magnetisation relaxation data for **3M**, **4M** and **5M**.

Supplementary Table 1. Relative energies of the seven 5f orbitals for the $[UN]^{2+}$ complex from *ab initio* calculations.

CSF	$\|l_z\|$	Relative energy (cm^{-1})
1	3	0
2	3	9
3	2	1301
4	2	1302
5	1	4787
6	1	4788
7	0	6270

Supplementary Table 2. Relative SO energies for the $[UN]^{2+}$ complex from *ab initio* calculations.

Doublet	Relative energy (cm^{-1})	j_z	g_{xy}	g_z
1	0	±5/2	0.00	4.20
2	1934	±3/2	0.00	2.26
3	5129	±1/2	2.14	0.65
4	6866	±7/2	0.01	8.00
5	7990	±5/2	0.01	5.80
6	10876	±3/2	0.00	3.74
7	12504	±1/2	4.14	1.35

Supplementary Table 3. Parameters derived from CF parameterisation of *ab initio* results for the $[UN]^{2+}$ complex.

**Parameter**	**Value (cm^{-1})**
B'^{0}_{2}	-211.8
B'^{0}_{4}	3.012
B'^{0}_{6}	0.008225
λ	2000

Supplementary Table 4. Relative energies of the seven 5f orbitals for the C_3 symmetrised $[UN(NH_3)(NH_2)_3]^{1-}$ complex from *ab initio* calculations.

CSF	$\lvert l_z \rvert$	Relative energy (cm^{-1})
1	2	0
2	2	1
3	3	43
4	3	1166
5	1	2176
6	1	2176
7	0	9649

Supplementary Table 5. Relative SO energies, and effective *g*-values, for the C_3 symmetrised $[UN(NH_3)(NH_2)_3]^{1-}$ complex from *ab initio* calculations.

Doublet	Relative energy (cm^{-1})	Approximate j_z	g_{xy}	g_z
1	0	±5/2	0.03	3.80
2	278	±3/2	0.00	2.26
3	3334	±1/2	1.53	0.10
4	6624	-	2.21	3.40
5	7436	-	2.17	5.25
6	8155	±3/2	0.00	3.59
7	14506	±1/2	3.51	1.89

Supplementary Table 6. Parameters derived from CF parameterisation of *ab initio* results for the C_3 symmetrised $[UN(NH_3)(NH_2)_3]^{1-}$ complex.

**Parameter**	**Value (cm^{-1})**
B'^0_2	-181.0
B'^0_4	7.128
B'^0_6	-0.7606
$\lvert B'^6_6 \rvert$	1.560

Supplementary Table 7. Parameters derived from CF parameterisation of *ab initio* results for the C_3 $[UN(NH_3)(NH_2)_3]^{1-}$ complex, including orbital reduction.

**Parameter**	**Value (cm^{-1})**
B'^0_2	-212.1
B'^0_4	9.610
B'^0_6	-1.335
$\lvert B'^6_6 \rvert$	2.571
λ	2195
κ	0.9201

Supplementary Table 8. Relative SO energies, and effective g-values, for **3M** from *ab initio* calculations.

**Doublet**	**3Li (U1)**				**3Li (U2)**			
	**Relative energy (cm^{-1})**	g_1	g_2	g_3	**Relative energy (cm^{-1})**	g_1	g_2	g_3
1	0	3.74	0.45	0.56	0	3.60	0.52	0.71
2	637	2.78	0.66	0.30	580	3.07	0.36	0.77
3	4281	0.30	1.85	1.67	4224	0.26	1.66	1.75
4	6743	0.80	1.68	5.27	6670	0.88	1.48	5.31
5	7696	0.93	1.58	6.10	7758	0.85	1.40	6.38
6	9421	3.94	0.03	0.22	9267	3.95	0.04	0.17
7	14613	1.80	3.75	3.61	14776	1.82	3.67	3.59

**Doublet**	**3Na**				**3K**			
	**Relative energy (cm^{-1})**	g_1	g_2	g_3	**Relative energy (cm^{-1})**	g_1	g_2	g_3
1	0	3.76	0.37	0.45	0	3.84	0.35	0.25
2	554	2.87	0.50	0.34	521	2.35	0.34	0.12
3	4299	0.28	1.71	1.62	4107	0.27	1.67	1.75
4	6682	0.98	1.66	5.07	6772	1.44	2.24	3.98
5	7629	1.00	1.60	6.19	7489	1.71	2.25	5.05
6	9254	3.88	0.04	0.20	9119	3.71	0.05	0.17
7	15243	1.83	3.56	3.60	14743	1.81	3.65	3.61

**Doublet**	**3Rb**				**3Cs**			
	**Relative energy (cm^{-1})**	g_1	g_2	g_3	**Relative energy (cm^{-1})**	g_1	g_2	g_3
1	0	3.84	0.43	0.34	0	3.87	0.27	0.22
2	527	2.60	0.49	0.27	523	2.59	0.31	0.36
3	4445	0.23	1.50	1.72	4351	0.21	1.48	1.72
4	6644	1.13	1.85	4.74	6664	4.61	1.19	1.91
5	7527	1.21	1.82	5.79	7526	5.71	1.48	1.68
6	9309	3.82	0.09	0.25	9220	3.77	0.13	0.32
7	15844	1.84	3.56	3.45	15786	1.84	3.46	3.57

Supplementary Table 9. Relative SO energies, and effective *g*-values, for **4M** from *ab initio* calculations.

**Doublet**	**4Na**				**4K**			
	**Relative energy (cm^{-1})**	g_1	g_2	g_3	**Relative energy (cm^{-1})**	g_1	g_2	g_3
1	0	3.86	0.11	0.20	0	3.88	0.27	0.22
2	824	2.13	0.21	0.12	794	2.28	0.35	0.13
3	4568	0.28	1.56	1.73	4617	0.21	1.47	1.65
4	6839	0.02	2.85	3.05	6750	0.24	2.40	4.29
5	7455	1.70	2.57	2.93	7488	1.15	2.09	4.57
6	9424	3.59	0.13	0.04	9403	3.73	0.03	0.21
7	16030	1.85	3.43	3.52	16628	1.87	3.36	3.46

**Doublet**	**4Rb**				**4Cs**			
	**Relative energy (cm^{-1})**	g_1	g_2	g_3	**Relative energy (cm^{-1})**	g_1	g_2	g_3
1	0	3.90	0.05	0.01	0	3.90	0.02	0.08
2	696	2.17	0.09	0.04	677	2.15	0.02	0.08
3	4510	0.20	1.63	1.48	4529	0.19	1.61	1.49
4	6765	1.01	2.90	2.79	6743	1.24	2.76	2.77
5	7412	3.23	2.37	2.64	7408	3.13	2.65	2.53
6	9248	3.60	0.05	0.00	9262	3.60	0.02	0.06
7	16561	1.87	3.39	3.44	16594	1.87	3.43	3.38

Supplementary Table 10. Relative SO energies, and effective *g*-values, for **5M** from *ab initio* calculations.

Doublet	5Li				5Na			
	Relative energy (cm^{-1})	g_1	g_2	g_3	Relative energy (cm^{-1})	g_1	g_2	g_3
1	0	3.83	0.34	0.24	0	3.88	0.08	0.03
2	639	2.73	0.43	0.24	656	2.12	0.04	0.02
3	4490	0.22	1.62	1.63	4634	0.31	1.70	1.76
4	6669	0.79	1.81	5.08	6765	1.79	2.65	2.68
5	7611	1.12	1.55	6.02	7461	3.56	2.40	2.45
6	9373	3.82	0.11	0.28	9601	3.60	0.01	0.02
7	15861	1.84	3.52	3.51	15685	1.83	3.53	3.56

Doublet	5K				5Rb			
	Relative energy (cm^{-1})	g_1	g_2	g_3	Relative energy (cm^{-1})	g_1	g_2	g_3
1	0	3.87	0.41	0.31	0	3.90	0.04	0.04
2	715	2.22	0.05	0.44	613	2.13	0.00	0.00
3	4636	0.33	1.62	1.77	4666	0.27	1.65	1.65
4	6792	0.92	2.25	4.21	6731	1.99	2.60	2.60
5	7476	1.55	2.10	4.70	7432	3.81	2.40	2.40
6	9585	3.76	0.12	0.00	9507	3.61	0.00	0.00
7	15732	1.83	3.60	3.49	16242	1.85	3.47	3.47

Doublet	5Cs			
	Relative energy (cm^{-1})	g_1	g_2	g_3
1	0	3.90	0.19	0.13
2	526	2.22	0.25	0.07
3	4570	0.19	1.62	1.50
4	6649	1.78	2.37	3.34
5	7418	1.83	2.39	4.75
6	9326	3.72	0.17	0.06
7	16598	1.86	3.46	3.39

Supplementary Table 11. Parameters derived from EPR with effective *s* = 1/2 model.

**Parameter**	**3K**	**3Rb**	**3Cs**
g_z	3.646	3.725	3.728
X-band linewidth (GHz)	0.3648	0.5816	0.6004
Q-band linewidth (GHz)	0.4176	0.7828	0.6128

Supplementary Table 11 cont. – Parameters derived from EPR with effective *s* = 1/2 model.

**Parameter**	**4Na**	**4Rb**	**4Cs**
g_z	3.770	4.057	3.715
X-band linewidth (GHz)	0.2576	0.3161	0.3921
Q-band linewidth (GHz)	0.2743	0.8241	0.6589

Supplementary Table 11 cont. – Parameters derived from EPR with effective *s* = 1/2 model.

**Parameter**	**5Li**	**5Na**	**5K**	**5Rb**	**5Cs**
g_z	3.741	3.747	3.739	3.737	3.708
X-band linewidth (GHz)	0.3720	0.2636	0.3878	0.2940	0.4750
Q-band linewidth (GHz)	0.4467	0.2965	0.4724	0.3853	0.2331

Supplementary Table 12. Positions of UV/Vis/NIR f-f transitions, in cm^{-1}.

**4Na**	**4K**	**4Rb**	**4Cs**
4600	4650	4650	4650
6063	6051	6045	6037
6923	6904	6904	6895
9031	8900	8850	8900
-	17544	17466	17650

Supplementary Table 12 cont. – Positions of UV/Vis/NIR f-f transitions, in cm^{-1}.

**5Li**	**5Na**	**5K**	**5Rb**	**5Cs**
4600	4750	4750	4750	4750
6055	6094	6072	6072	6059
6885	6891	6894	6894	6901
8900	9000	9019	9000	8900
18724	18181	17634	17303	17152

Supplementary Table 13. Relative SO energies, and effective *g*-values, for **4Na** from *ab initio* calculations with a larger basis set of VQZP/VTZP/VDZP quality *cf.* VTZP/VDZP/VDZ quality for Table S14.

Doublet	Relative energy (cm^{-1})	g_1	g_2	g_3
1	0	3.86	0.10	0.20
2	833	2.13	0.21	0.12
3	4562	0.29	1.57	1.74
4	6835	0.09	2.85	3.06
5	7447	1.64	2.56	2.92
6	9435	3.59	0.12	0.05
7	15974	1.85	3.53	3.43

Supplementary Table 13 cont. Relative SO energies, and effective *g*-values, for **4Na** from *ab initio* calculations with a larger active space of RAS1 = 3, RAS2 = 13, RAS3 = 3 (single excitations only) and CASPT2 corrections *cf.* RAS1 = 0, RAS2 = 7, RAS3 = 0 and no CASPT2 for Supplementary Table 14.

Doublet	Relative energy (cm^{-1})	g_1	g_2	g_3
1	0	3.92	0.10	0.25
2	1090	2.11	0.24	0.09
3	5620	1.70	1.18	0.40
4	6634	2.33	3.80	2.64
5	7537	3.03	0.56	2.51
6	10145	3.60	0.09	0.34
7	17742	1.83	3.28	3.06

Supplementary Table 14. Relative SO energies, and effective *g*-values, for [U(O)(Tren^{TIPS})] from *ab initio* calculations.

Doublet	Relative energy (cm^{-1})	g_1	g_2	g_3
1	0	2.27	0.12	0.08
2	683	3.89	0.16	0.12
3	4417	0.10	1.43	1.53
4	6648	4.80	1.26	1.30
5	8391	6.74	1.09	1.39
6	9101	3.70	0.05	0.16
7	16819	1.87	3.50	3.47

Supplementary Table 15. Parameters derived from EPR with simple effective s = 1/2 dimer model.

**Parameter**	**3Na**	**3K**	**3Rb**	**3Cs**	**4K**
g_z	3.640	3.646	3.725	3.725	3.723
$\lvert j_{zz} \rvert$	0.3681	< 0.0001	< 0.0051	< 0.0047	+0.0066
X-band linewidth (GHz)	1.2363	0.3648	0.3894	0.4612	0.2328
Q-band linewidth (GHz)	0.9828	0.4176	0.6791	0.5016	0.2111

Supplementary Table 16. Separation of the uranium ions, calculated dipolar interaction, and experimental estimate of the interaction using the s = 1/2 model, for compounds in series **3M**.

**Compound**	U···U distance (Å)	Dipolar j_{zz} (cm^{-1})	Observed $\lvert j_{zz}\rvert$ (cm^{-1})
**3Li**	6.95	$+0.0052 < j_{zz} < +0.018$	-
**3Na**	7.31	$+0.0044 < j_{zz} < +0.015$	0.3681
**3K**	7.77	$+0.0035 < j_{zz} < +0.012$	< 0.0001
**3Rb**	7.93	$+0.0034 < j_{zz} < +0.011$	< 0.0051
**3Cs**	8.23	$+0.0031 < j_{zz} < +0.011$	< 0.0047

Supplementary Table 17. Parameters derived from the temperature dependence of magnetisation relaxation.

Parameter	3Na	3K	3Rb	3Cs	4Na	4K	4Rb	4Cs
C (K^{-n} s^{-1})	0.366	0.217	1.11	6.41	0.0510	0.0239	0.220	0.193
n	7	7	6	5	7	7	6	6
${\tau_{QTM}}^{-1}$ (s^{-1})	286	-	-	-	19.8	2.98	-	-

Supplementary Table 17 cont. – Parameters derived from the temperature dependence of magnetisation relaxation.

Parameter	5Li	5Na	5K	5Rb	5Cs
C (K^{-n} s^{-1})	0.445	0.180	11.0	0.167	0.0276
n	6	6	4	6	7
${\tau_{QTM}}^{-1}$ (s^{-1})	-	-	812	-	-

Supplementary Note 1: ***Equations 1-5***

Through analytical solution to the axial CF Hamiltonian $\hat{H}_{CF(ax)} = B'^0_2\hat{O}^0_2 + B'^0_4\hat{O}^0_4 + B'^0_6\hat{O}^0_6$, the CFPs can be extracted from the orbital energies by solving the simultaneous equations below, where $E_{\pm 3} = 0$.

$$-15B'^0_2 - 600B'^0_4 - 1260B'^0_6 = E_{\pm 2} \quad (1)$$

$$-24B'^0_2 - 120B'^0_4 + 2520B'^0_6 = E_{\pm 1} \quad (2)$$

$$-27B'^0_2 + 180B'^0_4 - 3780B'^0_6 = E_0 \quad (3)$$

The analogous relations for d orbitals (l = 2), where all $B'^q_6 = 0$ and $E_{\pm 2} = 0$ are:

$$-9B'^0_2 - 60B'^0_4 = E_{\pm 1} \quad (4)$$

$$-12B'^0_2 + 60B'^0_4 = E_0 \quad (5)$$

Supplementary Note 2: ***General comments on new synthesis of uranium-nitrides***

We identified the limiting factor in the synthesis of **3Na** from **2** to be the low solubility of NaN_3 in pyridine, and since ethers and other polar solvents (e.g. acetonitrile, dichloromethane) react with **2** and **3Na**, such solvents cannot ameliorate this issue. Permutations of 12C4 addition alongside NaN_3 to increase the solubility of NaN_3 resulted in poorer yields, presumably due to sodium ion abstraction before it can stabilise the nitride installation, which has been shown to be key to preparing Tren^{TIPS}-supported terminal uranium nitrides.[1,2]

It is instructive to inspect the electron-transfer and bond-breaking/-forming chemistry that occurs in the synthesis of **3Na**:

$U^{III} + N_3^- + Na^I \rightarrow U^V{\equiv}N^- + Na^I + N_2$

Formally, the sodium ion has donated its valence electron to the azide, so the uranium(III) ion provides two electrons to effect ejection of dinitrogen and installation of a trianionic nitride. As well as the solubility issues of NaN_3, we surmised that having to establish a U-N bond during the reaction must provide decomposition opportunities. We postulated whether we could start with the azide group pre-coordinated to uranium, which would require uranium(IV) to avoid premature azide activation, and initiate dinitrogen elimination, one-electron oxidation of uranium, and nitride formation by the addition of an external one-electron reductant:

$U^{IV}\text{-}N_3 + M^0 \rightarrow U^V{\equiv}N^- + M^I + N_2$

Gratifyingly, methods based on this reaction give straightforward, reliable access to the DCIP series **3M** in respectable yields and permit us to greatly expand our uranium(V)-nitride family. Additional benefits include: (i) the synthesis of **7** has been improved (analytically pure yields of ~ 94%), giving higher yields of uranium(V)-nitrides overall, whereas the preparation of **2** is much less convenient; (ii) uranium(V)-nitrides with all alkali metal cations are now straightforwardly accessible, which may be desirable in subsequent reactions; (iii) avoiding the use of pyridine, the only solvent in which the prior route works, simplifies work-up and isolation.

We attribute the collective failure of multiple co-ligands to abstract lithium from **3Li** to a combination of the strongly nucleophilic nitride and the small, highly polarising lithium. This is consistent with the straightforward removal of the larger, softer heavier alkali metals from **3Na** – **3Cs** with the appropriate crown ethers to give the SIPs **4Na** – **4Cs**. When the size-matching is not optimised the nucleophilic nitride does not release the alkali metal and CIPs **5M** are isolated.

Supplementary Note 3: *Comments on structural analyses*

The metrical data for all of the complexes discussed here are largely as would be expected, but notably the U-N_{amine} distances in **3M**, **4M**, and **5M** are longer than for **1** [2.482(6) Å][3] and **6** [2.465(5) Å],[2] which has been attributed to the presence of an inverse-*trans*-influence (ITI);[2-4] on the basis of these bond lengths the ITI does not seem to be present in the uranium(V)-nitrides described here. There is no obvious trend in the **3M** series with respect to whether the $UN(M)_2NU$ cores are planar or *trans*-bent, but we suggest the observed geometries are modulated by crystal packing forces since the potential energy surfaces of the $UN(M)_2NU$ cores must be considered shallow. This is credible as the M-$N_{nitride}$ interactions will be electrostatic and thus easily perturbed. The grouping of the U≡N bond lengths of **3Li** – **3K** (long) versus **3Rb** and **3Cs** (short) can be rationalised on the basis that the former metals would be expected to polarise and lessen the electron density in the U≡N linkage more than the latter pair; the longest U≡N bond for **3K** may additionally be the result of the slight pyramidalisation of the nitride centre in this complex. Regarding the **5M** series, the metrical data for the U≡N-M angle most likely reflect the structural deformations required to enable the $\{M(crown)\}^+$ unit to optimise its approach to the $[U(Tren^{TIPS})(N)]^-$ fragment, but crystal packing forces must play a role; in support of this view, it is notable that cesium, which should present the most polar N-M bond, exhibits the most acute U-N-M angle.

Supplementary Methods

Experimental

General: All manipulations were carried out using Schlenk techniques, or an MBraun UniLab glovebox, under an atmosphere of dry nitrogen. Solvents were dried by passage

through activated alumina towers and degassed before use or were distilled from calcium hydride. All solvents were stored over potassium mirrors, except for ethers and pyridine which were stored over activated 4 Å sieves. Deuterated solvent was distilled from potassium, degassed by three freeze-pump-thaw cycles and stored under nitrogen. Sodium azide was dried under vacuum for four hours prior to use. Metallic sodium was purchased as 25-35 wt% dispersion in paraffin, washed with dry diethyl ether, dried under vacuum and stored at −30 °C in the glove box. Crown ethers were dissolved in ether, dried over activated 4 Å molecular sieves for 24 hours, decanted and the ether removed prior to use. $[U(Tren^{TIPS})(Cl)]$ $[Tren^{TIPS} = \{N(CH_2CH_2NSiPr^i_3)_3\}^{3-}$; $Pr^i = CH(CH_3)_2]$ was prepared as described previously.[1]

^{1}H and ^{29}Si NMR spectra were recorded on a Bruker 400 spectrometer operating at 400.2 and 79.5 MHz respectively; chemical shifts are quoted in ppm and are relative to TMS. UV/Vis/NIR spectra were recorded on a Perkin Elmer Lambda 750 spectrometer. Data were collected in 1 mm path length cuvettes loaded in an MBraun UniLab glovebox and were run versus the appropriate reference solvent. Note the f-f transition at ~4,700 cm^{-1} is partially obscured by imperfect background subtraction of solvent absorbance.

Variable-temperature (1.8 – 300 K) magnetic susceptibility (0.1 T dc field) and variable field (0-7 T) magnetisation data were recorded on a Quantum Design MPMS XL7 superconducting quantum interference device (SQUID) magnetometer equipped with a 7 T magnet. Ac susceptibility data were collected under either a 0.1 T dc field or a zero dc field, using a small ac field of 1.55 Oe oscillating at frequencies between 1 and 1400 Hz. For all measurements, doubly recrystallised powdered samples were carefully checked for purity and data reproducibility between several independently prepared batches for each compound examined. Care was taken to ensure complete thermalisation of the sample before each data point was measured and samples were immobilised in an eicosane matrix to prevent sample reorientation during measurements. Diamagnetic corrections were applied using tabulated Pascal constants and measurements were corrected for the effect of the blank sample holders (flame sealed Wilmad NMR tube and straw) and eicosane matrix. Solution magnetic moments were recorded at room temperature using the Evans method. Variable temperature EPR spectra were measured at X-band (ca. 9.4 GHz) and Q-band (ca. 34 GHz) on either a Bruker EMX 300 or a Bruker ElexSys E580 spectrometer equipped with an ER4119HS-W1 (X-band) or an ER 5106QT-E resonator. Gently ground polycrystalline samples were vacuum-sealed in 2.3 mm o.d. quartz tubes, to match both resonators. For X-band measurements, an additional 4 mm o.d. quartz tube was used as a support. Measurements were made on several independently prepared batches, which were also analysed by SQUID and CHN microanalyses, to ensure reproducibility. Spectra were background corrected against blank sample holders measured under identical conditions. In some samples we observe very weak, extremely sharp features in the $g \sim 2$ region which we believe are not intrinsic to the complexes; although the low-lying $j_z \approx \pm 3/2$ doublet (see main text) would have $g_z \approx 2$, the lineshapes would be absorption-like similar to the $g \sim 3.74(9)$ feature and thus are not consistent with such an assignment.

FTIR spectra were recorded on a Bruker Tensor 27 spectrometer. 50% ^{15}N-labelled analogues of **3**-**5** were prepared using sodium azide-1-^{15}N following procedures identical to those described below. We previously reported the observation of U≡N stretches in the FTIR spectra of **3Na** and **4Na** at ~930 cm^{-1} that shifted to ~900 cm^{-1} in the ^{15}N-labelled isotopomers, although the latter was complicated by overlapping bands from 12C4. An absorption at 936 cm^{-1} in the FTIR spectrum of SIP **4K** decreases in intensity by approximately 50% upon ^{15}N-labeling. This is accompanied by the growth of a band at 900 cm^{-1} for **^{15}N-4K**, giving an isotopomer shift of 36 cm^{-1}. This compares well to the calculated isotopomer shift to 906 cm^{-1} from reduced mass considerations. We assign these bands as U≡N stretches as they are identical to the bands and isotopomer shifts observed for **4Na** and similar values have been reported for uranium-nitride species prepared in matrix isolation experiments. However, we have been unable to conclusively identify isotopomer shifts for the remaining nitrides because of complex band structures in the fingerprint regions of their FTIR spectra. Attempts were made to record the Raman spectra of **3M**, **4M**, and **5M** but samples decompose in the beam (even at low power settings) and dilution experiments were inconclusive, precluding data acquisition and analysis.

Cyclic voltammetry experiments were attempted but these nitride complexes react with THF, chlorinated, and acetonitrile solvents and/or electrolytes so it was impracticable to collect electrochemical data.

CHN microanalyses were carried out by Tong Liu at the University of Nottingham. Some CHN data are persistently low, which is attributed to a combination of incomplete combustion from carbide/nitride formation, which has precedent with organo-silicon rich complexes,[5] but multiple measurements on multiple samples ensure the reliability of the data.

Preparation of [U(N)(Tren^{TIPS})] (6)

*Note: Due to the light sensitive nature of **6** all manipulations should be conducted in the absence of light.*

A solution of I_2 (60 mg, 0.24 mmol) in toluene (5 ml) was added dropwise over 20 minutes to a stirring solution of **4K** (0.64 g, 0.48 mmol) in toluene (10 ml) at –78 °C. The brown mixture was allowed to warm to room temperature with stirring over 16 h. Volatiles were removed *in vacuo* and the product was washed with pentane at –78 °C (2 × 5 ml). The solid was dried *in vacuo* to yield **6** as a red/brown powder and is stored in the dark at −30 °C. Yield: 0.24 g, 59%. The identity of **6** was confirmed by comparison to previously reported data.[2]

Improved preparation of [U(Tren^{TIPS})(N_3)] (7)

THF (40 ml) was added to a cold (−78 °C) stirring mixture of [U(Tren^{TIPS})(Cl)] (11.72 g, 13.20 mmol) and NaN_3 (1.45 g, 22.31 mmol). The mixture was allowed to warm to room temperature with stirring over 1 h and heated at 60 °C for 16 hrs. Once cool, volatiles were removed *in vacuo*. The product was extracted into hexanes (3 × 30 ml) at 60 °C and filtered

through a frit to remove the NaCl precipitate. The extracts were combined and volatiles removed *in vacuo* to yield a bright green/yellow solid. The product was dried *in vacuo* for 1 hr. Yield 11.12 g, 94%. The identity of **7** was confirmed by comparison to previously reported data.[2]

Attempts to prepare [U(Tren^{TIPS})(N)][Li(crown)$_2$] (4Li)

All attempts to prepare **4Li** were unsuccessful: we could not abstract the lithium cations from **3Li** to afford the SIP **4Li**. The list of potential abstracting ligands that failed to sequester lithium from **3Li** is long: benzo-9-crown-3, 12C4, 15C5, benzo-15-crown-5 (B15C5), 18-crown-6 (18C6), dibenzo-18-crown-6 (DB18C6), diethyl ether, tetrahydrofuran, pyridine, 4-dimethylaminopyridine, dimethoxyethane, N,N'-tetramethylethylenediamine, N,N',N"-pentamethyldiethylenetriamine, hexamethylphosphoramide, tetraglyme, pentaglyme, and [2.2.2]-cryptand.

***Ab initio method*:** State-averaged complete active space self-consistent field (CASSCF) calculations were performed using MOLCAS 7.8[6,7] employing the crystallographic geometries with no structural optimisation. Calculations for SIP complexes **4M** were performed on the uranium anions without the accompanying crown ether sequestered alkali metal cations, while all other species **3M** and **5M** were performed using the entire neutral molecule. In the case of the dimers **3M**, the crystallographically unique uranium sites were examined individually where the adjacent uranium ion was substituted with the diamagnetic protactinium(V). Unless otherwise stated, the ANO-RCC-VTZP, VTZ and VDZ basis sets[8,9] were used for the uranium ion, the first coordination sphere nitrogen atoms, and all other atoms, respectively. The two electron integrals were Cholesky decomposed using the default thresholds. The calculations employed the second order Douglas-Kroll-Hess (DKH) Hamiltonian, where scalar relativistic contractions are taken into account in the basis set and the spin-orbit coupling (SOC) is handled by diagonalising the SOC Hamiltonian in the basis of the CASSCF spin-free states with the RASSI module. Unless otherwise stated, the active space consisted of one electron in the seven 5f orbitals of uranium, where all seven doublet states were considered in the state-averaged CASSCF orbital optimisation, as well as in the subsequent SO mixing.

Supplementary References

1. King, D. M., Tuna, F., McInnes, E. J. L., McMaster, J., Lewis, W., Blake, A. J., Liddle, S. T. Synthesis and Structure of a Terminal Uranium Nitride Complex *Science* **337**, 717-720 (2012).
2. King, D. M., Tuna, F., McInnes, E. J. L., McMaster, J., Lewis, W., Blake, A. J., Liddle, S. T. Isolation and characterisation of a uranium(VI)-nitride triple bond *Nat. Chem.* **5**, 482-488 (2013).

3. King, D. M., Tuna, F., McInnes, E. J. L., McMaster, J., Lewis, W., Blake, A. J., Liddle, S. T. Single-Molecule Magnetism in a Single-Ion Triamidoamine Uranium(V) Terminal Mono-Oxo Complex. *Angew. Chem. Int. Ed.* **52**, 4921–4924 (2013).
4. Denning, R. G. Electronic Structure and Bonding in Actinyl Ions and their Analogs. *J. Phys. Chem. A* **111**, 4125–4143 (2007).
5. Hitchcock, P. B., Lappert, M. F., Maron, L. & Protchenko, A. V. Lanthanum does form stable molecular compounds in the +2 oxidation state. *Angew. Chem. Int. Ed.* **47**, 1488-1491 (2008).
6. Aquilante, F., De Vico, L., Ferré, N., Ghigo, G., Malmqvist, P., Neogrády, P., Pedersen, T. B., Pitoňák, M., Reiher, M., Roos, B. O., Serrano-Andrés, L., Urban, M., Veryazov, V., Lindh, R. MOLCAS 7: The Next Generation. *J. Comput. Chem.* **31**, 224-247 (2010).
7. Aquilante, F., Autschbach, J., Carlson, R. K., Chibotaru, L. F., Delcey, M. G., De Vico, L., Fdez. Galván, I., Ferré, N., Frutos, L. M., Gagliardi, L., Garavelli, M., Giussani, A., Hoyer, C. E., Li Manni, G., Lischka, H., Ma, D., Malmqvist, P. Å., Müller, T., Nenov, A., Olivucci, M., Pedersen, T. B., Peng, D., Plasser, F., Pritchard, B., Reiher, M., Rivalta, I., Schapiro, I., Segarra-Martí, J., Stenrup, M., Truhlar, D. G., Ungur, L., Valentini, A., Vancoillie, S., Veryazov, V., Vysotskiy, V. P., Weingart, O., Zapata, F., Lindh, R. MOLCAS 8: New Capabilities for Multiconfigurational Quantum Chemical Calculations Across the Periodic Table. *J. Comput. Chem.* **37**, 506-541 (2016).
8. Roos, B. O., Lindh, R., Malmqvist, P.-Å., Veryazov, V., Widmark, P. -O. Main Group Atoms and Dimers Studied with a New Relativistic ANO Basis Set. *J. Phys. Chem. A* **108**, 2851-2858 (2004).
9. Roos, B. O., Lindh, R., Malmqvist, P.-Å., Veryazov, V., Widmark, P. -O. New Relativistic ANO Basis Sets for Actinide Atoms. *Chem. Phys. Lett.* **409**, 295-299 (2005).
